# Very low prevalence of *Opisthorchis viverrini* s.l. cercariae in *Bithynia siamensis siamensis* snails from the canal network system in the Bangkok Metropolitan Region, Thailand

**DOI:** 10.1051/parasite/2020072

**Published:** 2021-01-08

**Authors:** Phuphitchan Rachprakhon, Watchariya Purivirojkul

**Affiliations:** Animal Systematics and Ecology Speciality Research Unit, Department of Zoology, Faculty of Science, Kasetsart University Bang Khen Campus 10900 Bangkok Thailand

**Keywords:** Prevalence, Cercarial infection, Liver fluke, Digenean larva, Intermediate host, Flowing-water habitat

## Abstract

The liver fluke *Opisthorchis viverrini* s.l. is associated with a long-term public health problem in Thailand. However, *O. viverrini* s.l. infection in *Bithynia* snails in the canal network system (CNS) in the Bangkok Metropolitan Region (BMR) has never been assessed. This study aimed to investigate the occurrence of *B. siamensis siamensis* and the prevalence of *O. viverrini* s.l. infection in this snail in the CNS in BMR along with morphological examination and molecular analyses on *O. viverrini* s.l. cercariae. The snails were randomly sampled from the CNS in all BMR areas from January 2018 to July 2019. Snail specimens were identified and examined for digenean infection by shedding and dissection. The cercariae were identified using morphology and molecular methods, including PCR with a species-specific primer and a Bayesian phylogenetic analysis of ITS2 sequences. *Bithynia siamensis siamensis* was found in almost all sampling localities, with different quantities and detected frequencies. From a total of 7473 *B. s. siamensis* specimens, *O. viverrini* s.l. infections were detected in the Northern Bangkok, Muang Nakhon Pathom, Krathum Baen, and Lam Luk Ka areas with an overall prevalence of 0.05% (4/7473) and prevalence of 0.22% (1/455), 0.21% (1/469), 0.40% (1/253), and 0.16% (1/614) in individual localities with positive snails, respectively. This study is the first investigation of digenean infection in the canal network system-type habitat in Thailand and revealed extremely low *O. viverrini* s.l. prevalence.

## Abstract (fr)

La douve du foie *Opisthorchis viverrini* s.l. cause un problème de santé publique récurrent en Thaïlande. L’infection des *Bithynia* par *O. viverrini* s.l. dans le réseau de canaux (RC) dans la région métropolitaine de Bangkok (RMB) n’a jamais été évaluée. Cette étude visait à étudier la présence de *B. siamensis siamensis* et la prévalence de l’infection par *O. viverrini* s.l. chez ce mollusque dans le RC de la RMB, avec aussi une étude morphologique et des analyses moléculaires sur les cercaires d’ *O. viverrini* s.l. Les mollusques ont été prélevés au hasard dans le RC dans toutes les zones de la RMB de janvier 2018 à juillet 2019. Les spécimens de mollusques ont été identifiés et l’infection par les Digènes a été étudiée par excrétion et dissection. Les cercaires ont été identifiées à l’aide de méthodes morphologiques et moléculaires, y compris la PCR avec une amorce spécifique à l’espèce et une analyse phylogénétique bayésienne des séquences ITS2. *Bithynia siamensis siamensis* a été trouvé dans presque toutes les localités d’échantillonnage, avec des quantités et des fréquences de détection différentes. Sur un total de 7473 spécimens de *B. s. siamensis*, des infections par *O. viverrini* s.l. ont été détectées dans les régions du nord de Bangkok, Muang Nakhon Pathom, Krathum Baen et Lam Luk Ka avec une prévalence globale de 0,05 % (4/7473) et une prévalence respective de 0,22 % (1/455), 0,21 % (1/469), 0,40 % (1/253) et 0,16 % (1/614) dans les localités avec des mollusques positifs. Cette étude est la première enquête sur l’infection des Digènes dans l’habitat de type réseau de canaux en Thaïlande et a révélé une très faible prévalence d’*O. viverrini* s.l.

## Introduction

The fish-borne zoonotic digenean, *Opisthorchis viverrini* sensu lato (s.l.) has caused a significant, sustained public health problem in the Lower Mekong Basin [24]. In Thailand, opisthorchiasis is most prevalent in the northeastern region, decreasing in the northern, central, and southern regions, respectively [55]. *Opisthorchis viverrini* s.l. utilizes three snail taxa, i.e., *Bithynia funiculata*, *B. siamensis goniomphalos*, and *B. s. siamensis*, as the first intermediate host, and central Thailand has broad distribution of *B. s. siamensis* [23]. The cercarial stage and *Bithynia* snails play a pivotal role in spreading *O. viverrini* s.l. in ecosystems and limiting the opisthorchiasis epidemic boundaries [18, 26]. Moreover, prevalence data of *O. viverrini* s.l. infection in snails reflect the transmission of *O. viverrini* s.l. from definitive hosts to the environment [7, 32], and are useful for forecasting the epidemiological situation of infections in fishes and humans for the development of preventive applications for the future [1, 55].

Although Bangkok Metropolitan Region (BMR) is a part of central Thailand, it has two unique characteristics that might be of epidemiological significance. First, BMR is the most densely populated area in Thailand and has the most migrant workers from other regions and neighboring countries [5]. Second, the canal network system (CNS) in BMR, which is the most comprehensive and dense CNS in Thailand [33, 50], is possibly an ideal habitat for transmissions and dispersions of digeneans throughout their life cycles since: (1) it might have high contamination of helminths [61]; (2) it is a never-dry, aquatic habitat [44, 50] in which abundance and diversity of snail and fish hosts could be high all year round [34]; (3) it has excellent distribution routes for aquatic animals [15]; and (4) it is a public water source where people can catch fish hosts. These might reflect the epidemiological importance of the CNS. Nevertheless, BMR is a poorly and only very partially studied area concerning digenean infection in freshwater snails [1, 59]. Furthermore, *O. viverrini* s.l. infection in any *Bithynia* taxa, including *B. s. siamensis*, and their occurrences in any of Thailand’s CNSs have not been investigated to date.

To assess the epidemiological situation of *O. viverrini* s.l. infection in *B. s. siamensis* in the CNS of BMR, the occurrence of *B. s. siamensis* and the prevalence of *O. viverrini* s.l. infection in this snail were investigated. In addition, morphological and molecular analyses were performed to identify and describe *O. viverrini* s.l. cercariae obtained from this uninvestigated *O. viverrini* special habitat type in the area unconnected to the Mekong River and its tributaries.

## Materials and methods

### Ethics statement

The ethics of using animals to nurture the collected snail specimens and the investigation of *O. viverrini* s.l. infection in this study were approved by the ethics committee at Kasetsart University (Approval No. ACKU61-SCI-034).

### Snail specimen collection

**Figure 1:**
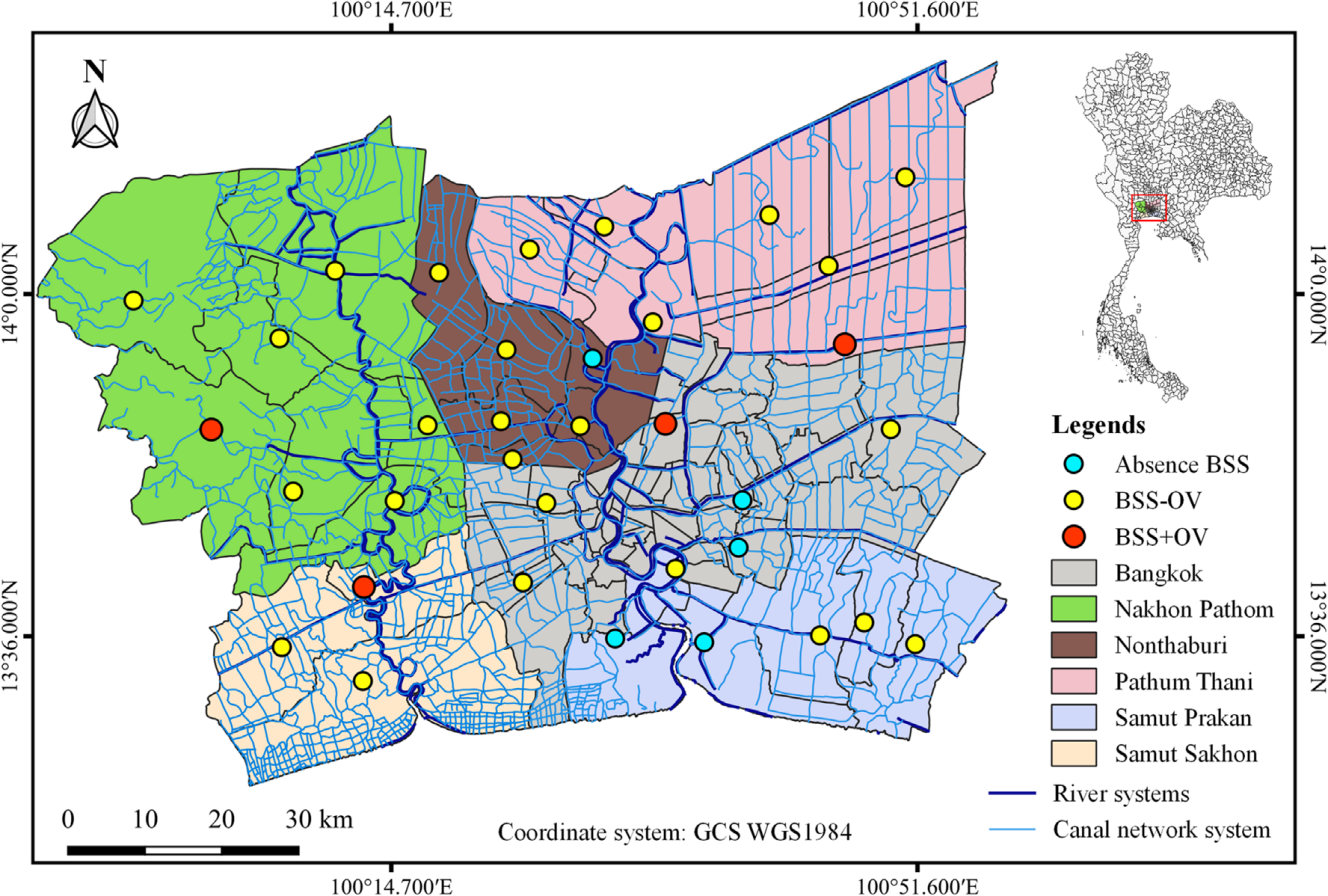
Map of study area in the Bangkok Metropolitan Region, Thailand. Sky blue spot, yellow spot, and red spot represent: (1) sampling locality without distribution of *Bithynia siamensis siamensis* snails, (2) sampling locality with *B. s. siamensis* distribution but no detected *Opisthorchis viverrini* s.l., and (3) locality with *B. s. siamensis* distribution and detected *O. viverrini* s.l., respectively.

Thirty-five localities of the CNS in BMR’s six provinces were chosen as snail-sampling localities; there was one sampling locality per district/area boundary (Fig. 1 and Table 1). The geographical coordinates in WGS 1984 datum for each sampling locality were marked by the global positioning system. The random samplings of *B. s. siamensis* from canals were conducted using one collector and 20 min at each locality by hand-picking and scooping based on the counts per unit of sampling time method [38] every three months from January 2018 through July 2019. The collected snails were identified using shell morphological criteria according to the taxonomic keys [8].

**Table 1:** GenBank accession numbers of ITS2 sequence data used for phylogenetic analysis.

Taxon		Accession no.
Ingroup		
Opisthorchiidae	OVCLLK18	MT781433
Opisthorchiidae	*Opisthorchis viverrini*	AY584735.1
Opisthorchiidae	*Opisthorchis viverrini*	HQ328548.1
Opisthorchiidae	*Opisthorchis viverrini*	HQ328549.1
Opisthorchiidae	*Opisthorchis viverrini*	HQ328550.1
Opisthorchiidae	*Opisthorchis viverrini*	KT894940.1
Opisthorchiidae	*Opisthorchis viverrini*	KT894941.1
Opisthorchiidae	*Opisthorchis viverrini*	KT894942.1
Opisthorchiidae	*Opisthorchis viverrini*	KT894943.1
Opisthorchiidae	*Opisthorchis viverrini*	KF577570.1
Opisthorchiidae	*Opisthorchis viverrini*	KF577571.1
Opisthorchiidae	*Opisthorchis viverrini*	KT726408.1
Opisthorchiidae	*Opisthorchis viverrini*	KT726408.1
Opisthorchiidae	*Opisthorchis noverca*	KC109193.1
Opisthorchiidae	*Opisthorchis felineus*	DQ513404.1
Opisthorchiidae	*Opisthorchis pedicellata*	KU688153.1
Opisthorchiidae	*Opisthorchis sudarikovi*	MK227161.1
Outgroup		
Opisthorchiidae	*Clonorchis sinensis*	JQ048601.1
Opisthorchiidae	*Metorchis bilis*	MG952282.1
Opisthorchiidae	*Metorchis orientalis*	MT231323.1
Opisthorchiidae	*Metorchis ussuriensis*	KP222497.1
Opisthorchiidae	*Metorchis xanthosomus*	KT740983.1
Opisthorchiidae	*Erschoviorchis anuiensis*	MK877248.1
Heterophyidae	*Metagonimus yokogawai*	KJ631734.1
Heterophyidae	*Heterophyes heterophyes*	KX431325.1
Heterophyidae	*Centrocestus formosanus*	KY075663.1
Heterophyidae	*Haplorchis yokogawai*	HM004160.1
Heterophyidae	*Haplorchis pumilio*	KX815125.1
Heterophyidae	*Haplorchis taichui*	KX815126.1

### Examinations of *O. viverrini* s.l. infection

The *B. s. siamensis* specimens were examined for *O. viverrini* s.l. infections by cercarial shedding and dissection. Each snail individual was placed in a small transparent plastic cup containing de-chlorinated water, and then exposed to light with an intensity of approximately 3000 lx from daylight-LED tube lights (850 lm) from 6:00 AM to 2:00 PM at room temperature (25 ± 2 °C). Subsequently, each cup was examined under a dissecting microscope. The living cercariae were fixed with 10% neutral buffered formalin (NBF), and some were stained with 0.5% neutral red dye (NR). They were then investigated and photographed with a brightfield Olympus BX51 fitted to an Olympus DP70 digital camera (Olympus Corporation, Japan). Afterward, snails were dissected, then investigated in the same manner as the shedding procedure described above.

### Morphological identification and descriptive study of *O. viverrini* s.l. cercariae

The photomicrographs of mature-cercarial specimens were utilized to identify the *O. viverrini* s.l species according to the morphological descriptions [23, 45, 63]. Measurements of 17 morphological characteristics (Table 4 and Fig. 4C) of the 20 photographed *O. viverrini* s.l. cercariae (from all four infected snails) fixed with 10% NBF, were performed in ImageJ version 1.50e. Metric data in micrometers (μm) of all measured characteristics in the text and Table 4 are represented as the arithmetic mean ± standard deviation followed by range (minimum–maximum) values in parentheses. The multi-planar photomicrographs of *O. viverrini* s.l. cercariae were used in drawing and labeling to display their characteristic details (Fig. 4).

### DNA preparation

Approximately 1200 individuals of the morphologically authenticated *O. viverrini* s.l. cercaria were collected separately from each infected-*B. s. siamensis* individual (the total number of infected snails was 4), washed with phosphate buffered saline and ultrapure water, and preserved in absolute ethanol within each 1.5 mL microcentrifuge tube. For use as a comparator in molecular detection, the *O. viverrini* s.l. adults preserved in 70% ethanol were obtained from the Faculty of Medicine at Khon Kaen University, Thailand. The genomic DNA of the cercarial and adult specimens was extracted separately using a GF-1 Tissue DNA Extraction Kit (Vivantis, Malaysia), according to the manufacturer’s protocol.

### Molecular detection using species-specific primers

**Figure 2:**
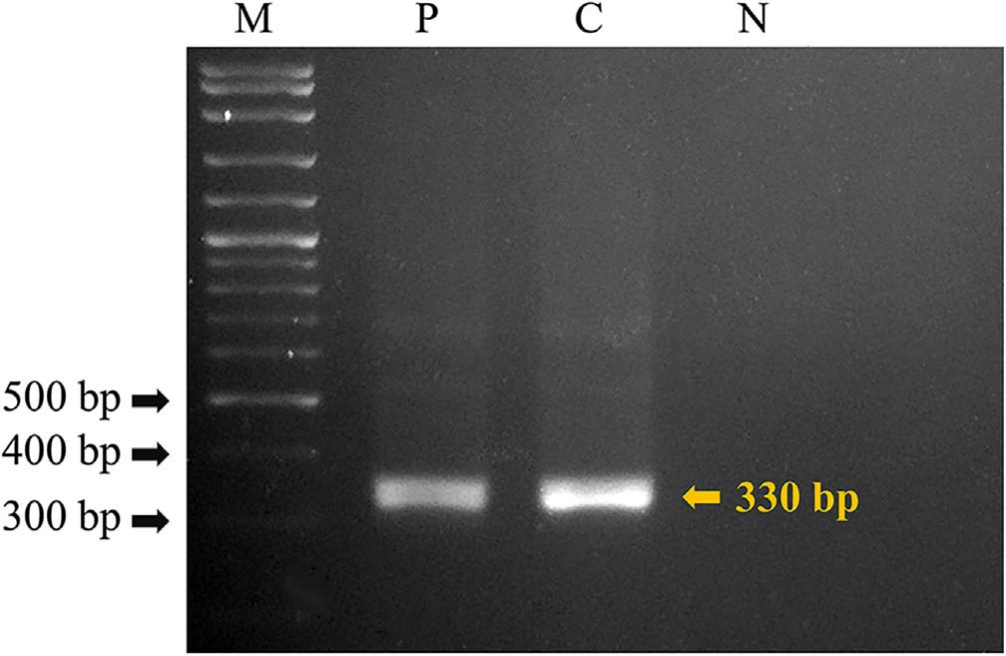
Agarose gel (2%) showing specific bands of 330 bp achieved by the pOV-A6 primers for *Opisthorchis viverrini* s.l. Lane M: 100 bp DNA markers, lane P: *O. viverrini* adult (positive control), lane C: *O. viverrini* s.l. cercaria from *Bithynia siamensis siamensis* snails, and lane N: ultrapure water (negative control).

PCR amplification of a 330 bp specific region using a pOV-A6 probe was performed four times for the 4 DNA samples of *O. viverrini* s.l. cercariae and the DNA sample of *O. viverrini* s.l. adults with the OV-6F and OV-6R primers from Wongratanacheewin et al. [62] in a total reaction volume of 50 μL containing 1× PCR buffer (ViBuffer A, Vivantis, Malaysia), 2 mM MgCl_2_, 0.7 μM of each primer, 1 mM dNTP mixture, 1 μL *Taq* DNA polymerase (5 u/μL), and 10 μL DNA template in a thermal cycler (Mastercycler Pro, Eppendorf, Germany), following the operating conditions of Prasopdee et al. [42]. Gel electrophoresis was operated to investigate a 330 bp band of PCR products in 2% agarose gel. PCR products of *O. viverrini* s.l. adults and ultrapure water were used as positive and negative controls (Fig. 2).

### ITS2 amplification

One from a total of 4 DNA samples of cercariae, which were all verified as the *O. viverrini* s.l. species with molecular detection, was used as a representative to analyze further by phylogenetic analysis. The ITS2 region was amplified by PCR with the ITS3 and ITS4 primers from Barber et al. [3] in a total reaction volume of 50 μL containing 1× PCR buffer (ViBuffer A, Vivantis, Malaysia), 2 mM MgCl_2_, 0.4 μM of each primer, 0.16 mM dNTP mixture, 0.4 μL Taq DNA polymerase (5 u/μL), and 2 μL DNA template in a thermal cycler (Mastercycler Pro, Eppendorf, Germany) with the following operating conditions: initial denaturation at 94 °C for 5 min, followed by 35 cycles of denaturation at 94 °C for 1 min each, annealing at 56 °C for 1 min, and extension at 72 °C for 30 s, then followed by a final extension at 72 °C for 10 min. PCR products were inspected using gel electrophoresis in the same manner as molecular detection. The amplified DNA sample was sent to Macrogen (Korea) to purify, and the sequencing was then performed using ITS3 and ITS4 primers.

### Molecular identification and phylogenetic analysis

The forward and reverse DNA chromatograms and sequences were processed in BioEdit version 7.2.5 to obtain a consensus sequence OVCLLK18 (Table 1), then submitted to the GenBank database. A dataset of 28 comparative ITS2 sequences was retrieved from GenBank (Table 1). All sequences were aligned by ClustalW and improved by being trimmed in BioEdit version 7.2.5. The GTR + G model was the best-fit model of nucleotide substitution for the sequence dataset by evaluating with MrModeltest version 2.4 via PAUP* version 4.0a using the Akaike Information Criterion. For phylogenetic analysis using Bayesian Inference (BI), *Haplorchis taichui* in the Heterophyidae family (accession number KX815126.1 in Table 1) was determined as an outgroup. BI analysis was executed in MrBayes version 3.2.7a using four chains of a Markov chain Monte Carlo algorithm for 15 million generations, with a sample frequency of 100. The first 25% of the sample trees were discarded, and the remaining sample trees were used to build a 50% majority-rule consensus tree and calculate Bayesian posterior probabilities (BPPs).

## Results

### Occurrences of *B. s. siamensis* snails

A total of 7473 *B. s. siamensis* were detected from almost all the sampling localities, except South Bangkok, Central Bangkok, Pak Kret, Phra Samut Chedi, and Muang Samut Prakan (Fig. 1 and Table 2). Each locality had different detection frequencies and various quantities of *B. s. siamensis* (Table 2). The *B. s. siamensis* were found in all localities of only Nakhon Pathom Province, along with the highest frequency of detecting *B. s. siamensis*.

**Table 2:** Occurrences of *Bithynia siamensis siamensis* and *Opisthorchis viverrini* s.l., and prevalences of *O. viverrini* s.l. infection in each sampling locality in the Bangkok Metropolitan Region, Thailand.

Province	District/Area	Latitude	Longitude	*B. s. siamensis*			*O. viverrini* s.l.		
				Detected frequency (Total *n* = 7)	Total no. of snails encountered	Total no. of infected snails	Detected frequency (Total *n* = 7)	Prevalence at detected time	Total prevalence
BK	North Thonburi	13°45′21.2″ N	100°25′34.0″ E	6	62	0	0	0	0
BK	South Thonburi	13°39′46.7″ N	100°23′55.8″ E	1	4	0	0	0	0
*BK	South Bangkok	13°42′13.7″ N	100°39′03.0″ E	0	0	0	0	0	0
*BK	Central Bangkok	13°45′33.2″ N	100°39′19.4″ E	0	0	0	0	0	0
BK	East Bangkok	13°50′31.7″ N	100°49′43.6″ E	7	347	0	0	0	0
**BK	North Bangkok	13°50′52.0″ N	100°34′21.5″ E	7	455	1	1	1.16	0.22
NP	Bang Len	14°01′38.4″ N	100°10′45.7″ E	7	51	0	0	0	0
NP	Kamphaeng Saen	13°59′33.1″ N	99°56′37.4″ E	1	1	0	0	0	0
NP	Don Tum	13°56′53.8″ N	100°06′51.3″ E	7	309	0	0	0	0
**NP	Muang^NP^	13°50′30.1″ N	100°02′04.9″ E	6	469	1	1	2.17	0.21
NP	Nakhon Chai Sri	13°46′09.7″ N	100°07′49.0″ E	7	331	0	0	0	0
NP	Sam Phran	13°45′29.8″ N	100°14′56.4″ E	7	230	0	0	0	0
NP	Phutthamonthon	13°50′47.9″ N	100°17′14.6″ E	6	300	0	0	0	0
*NB	Pak Kret	13°55′29.2″ N	100°28′49.1″ E	0	0	0	0	0	0
NB	Bang Bua Thong	13°56′05.6″ N	100°22′46.4″ E	6	169	0	0	0	0
NB	Sai Noi	14°01′30.6″ N	100°18′02.8″ E	7	568	0	0	0	0
NB	Bang Yai	13°51′05.1″ N	100°22′23.3″ E	7	722	0	0	0	0
NB	Muang^NB^	13°50′44.8″ N	100°27′56.9″ E	1	1	0	0	0	0
NB	Bang Kruai	13°48′24.6″ N	100°23′11.9″ E	5	44	0	0	0	0
SS	Krathum Baen	13°39′27.9″ N	100°12′46.8″ E	7	253	1	1	4.55	0.40
SS	Ban Phaew	13°35′14.3″ N	100°07′01.4″ E	6	233	0	0	0	0
SS	Muang^SS^	13°32′52.0″ N	100°12′42.4″ E	1	1	0	0	0	0
*SP	Phra Samut Chedi	13°35′50.8″ N	100°30′23.7″ E	0	0	0	0	0	0
SP	Phra Pradaeng	13°40′44.5″ N	100°34′38.0″ E	1	1	0	0	0	0
*SP	Muang^SP^	13°35′35.1″ N	100°36′38.3″ E	0	0	0	0	0	0
SP	Bang Phli	13°36′03.6″ N	100°44′46.0″ E	4	11	0	0	0	0
SP	Bang Sao Thong	13°36′58.0″ N	100°47′51.7″ E	7	156	0	0	0	0
SP	Bang Bo	13°35′27.1″ N	100°51′26.5″ E	5	112	0	0	0	0
**PT	Lam Luk Ka	13°56′29.0″ N	100°46′30.1″ E	7	614	1	1	0.85	0.16
PT	Thanyaburi	14°01′58.8″ N	100°45′21.7″ E	7	391	0	0	0	0
PT	Nong Sua	14°08′12.1″ N	100°50′45.0″ E	6	198	0	0	0	0
PT	Khlong Luang	14°05′32.9″ N	100°41′12.7″ E	7	1196	0	0	0	0
PT	Sam Khok	14°04′44.4″ N	100°29′37.8″ E	2	4	0	0	0	0
PT	Lat Lum Kaew	14°03′07.9″ N	100°24′24.1″ E	7	238	0	0	0	0
PT	Muang^PT^	13°58′01.6″ N	100°33′02.4″ E	1	2	0	0	0	0
Overall total				156	7473	4	4	1.47	0.05

Rows with one asterisk (*) and two asterisks (**) at the header row represent a sampling locality without *B. s. siamensis* distribution and a sampling locality with detected *O. viverrini* s.l. infection, respectively. Abbreviations: BK = Bangkok, NP = Nakhon Pathom, NB = Nonthaburi, SS = Samut Sakhon, SP = Samut Prakan, PT = Pathum Thani.

### Occurrences and prevalences of *O. viverrini* s.l.

*Opisthorchis viverrini* s.l. cercariae were detected in four individuals of *B. s. siamensis*, only using the cercarial shedding method (Fig. 1 and Table 2). The overall prevalence of *O. viverrini* s.l. infections detected in the North Bangkok, Muang Nakhon Pathom, Krathum Baen, and Lam Luk Ka areas was 0.05% (4/7473). Only one *O. viverrini* s.l.-infected snail was found one time in each infected locality, which represented a prevalence at the time of infection of 1.16% (1/86), 2.17% (1/46), 4.55% (1/22), and 0.85% (1/118), respectively, and a total prevalence of 0.22% (1/455), 0.21% (1/469), 0.40% (1/253), and 0.16% (1/614) in each infected locality, respectively (Table 2). Mean *O. viverrini* s.l. prevalence was 0.22% (0.16–0.40%). The prevalence of other cercarial types is shown in Table 3.

**Table 3:** Prevalence of other cercarial types detected in *Bithynia siamensis siamensis* snails in this study.

Cercarial type	Taxon	Prevalence	2nd intermediate host	Definitive host
Parapleurolophocercous	Heterophyidae	0.28% (21/7473)	Freshwater fishes [45]	Avians, mammals [39, 45]
Monostome	Notocotylidae	0.19% (14/7473)	Non-living objects*, vegetations [39]	Avians, mammals [45]
Cystophorous	Hemiuridae	0.32% (24/7473)	Microcrustaceans [39, 45]	Freshwater fishes, amphibians [39, 45]
Xiphidiocercaria (2 subtypes)	Lecithodendriidae	4.23% (316/7473)	(Aquatic insects and their larvae, crustaceans) [39, 45]	Amphibians, avians, bats [45]
	Plagiorchiidae		Tadpoles [39, 45]	All vertebrate classes [39, 45]
Furcocercous (3 subtypes)	Sanguinicolidae	0.63% (47/7473)	Not required [39, 45]	Freshwater fishes [39, 45]
	Strigeidae		Freshwater snails and fishes, frogs, [39, 45]	Avians, mammals [39, 45]
	Cyathocotylidae		Freshwater fishes [45]	Reptiles, avians, mammals [45]
Cercariaeum	Lissorchiidae	3.24% (242/7473)	Freshwater snails [49]	Freshwater fishes [49]

* Based on our observations during shedding times. Parentheses refer to the second intermediate host of both taxa.

### Morphological description of *O. viverrini* s.l. cercariae

**Figure 3:**
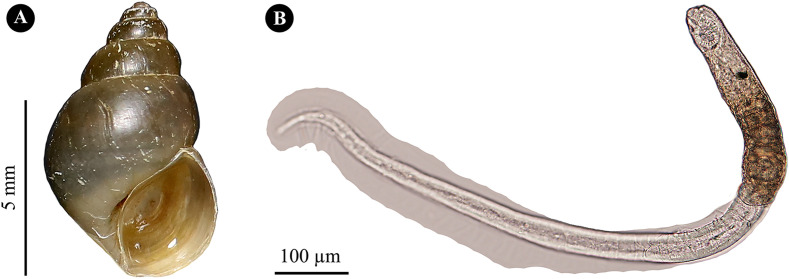
(A) Live *Bithynia siamensis siamensis* snail and (B) unstained live free-swimming *O. viverrini* s.l. cercaria in the bent-billiard tobacco pipe form at resting and fleetingly hanging times.

**Figure 4:**
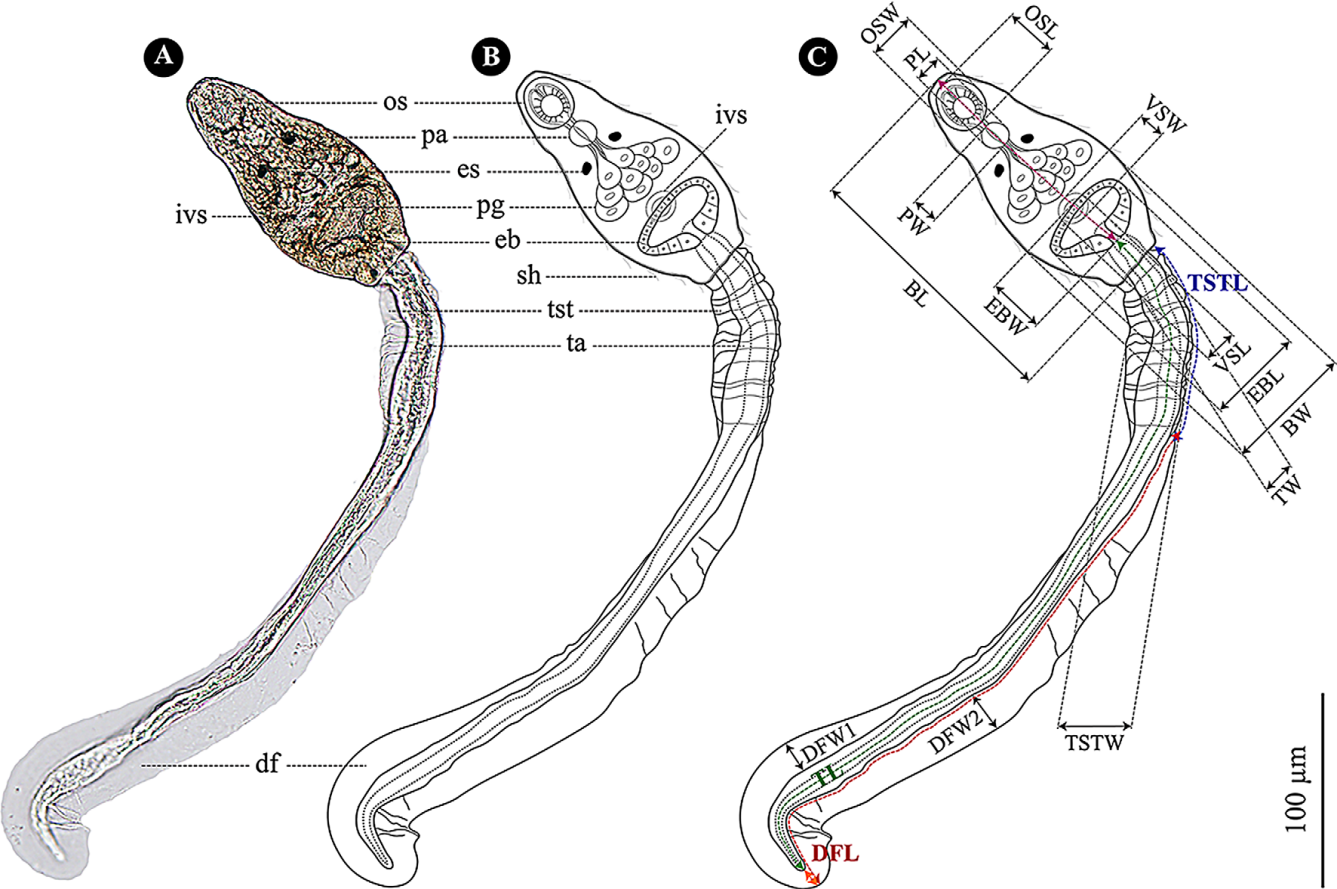
Morphology of *Opisthorchis viverrini* s.l. cercariae. (A) Photomicrograph of unstained cercaria fixed in 10% NBF, (B) drawing image, (C) measured morphological characters. Abbreviations in (A) and (B): os = oral sucker, pa = pharynx, es = eye spot, pg = penetration gland, ivs = inconspicuous ventral sucker, eb = excretory bladder, sh = sensory hair, tst = transversely striated tegument, ta = tail, df = dorso-ventral finfold. The definition of abbreviations in (C) was showed in the first column of Table 4. Total length = length of purple dashed arrow + TL + length of orange arrow (at the position following the end of the tail).

The mature free-swimming *O. viverrini* s.l. cercariae were the bent-billiard tobacco pipe form, concaving on the ventral side and bent outward on the dorsal side at the time of cercarial resting and momentarily hanging in the water (Fig. 3B). Its main structures consisted of a body and tail (Figs. 3 and 4). The body had uneasily visible sensory hairs on the surface, was ovate, and had prominent brownish granular pigments that spread bilaterally and symmetrically over the body. The obvious ovate muscular oral sucker located near the midway anterior end of the body possessed three rows of molar tooth-like structures that were quite noticeable when the oral sucker contracted. The ventral sucker located in front of the excretory bladder was small and nearly unnoticeable. Each of 10 penetration glands was a slightly evident translucent sac-like structure and possessed one duct. These ducts located at the median line of the body came out of the glands, passed through the central gap between one pair of the striking dark-brown pigmented eye spots, passed over the less apparent spherical pharynx, continued to the oral sucker, and finally opened toward the orifice of the oral sucker. There were numerous cystogenous glands with a small, relatively flat circular droplet-like shape that spread in dorso-ventral and anterior–posterior directions on both sides of the body, which were easily visible for the stained specimens.

The excretory bladder had a thick wall, especially in the posterior position, and was large and clearly discernible. The shape thereof was different according to the specimen’s state: globular in free-swimming specimens, especially in the bent-billiard tobacco-pipe shaped cercariae; an inverted triangle with rounded corners in the alive stained and fixed unstained specimens; and trapezoidal with rounded corners in fixed unstained specimens. At the anterior position close to the excretory bladder wall, a genital primordium was a nearly flattened translucent triangular lump with a base, which has a base and height equal to 4/5 and 1/5 of the bladder length. The genital primordium was effortlessly seen in unfixed stained and fixed unstained specimens if it was neither concealed nor pushed down to the excretory bladder’s dorsal location by the glandular penetration sacs when the anterior part of the cercarial body shrinks.

The tail’s origin was connected to the posterior end of the excretory bladder within the body. The tail was a long translucent cylindrical structure with a circumference that was gradually reduced from the widest anterior tail portion to the smallest portion at the posterior tip. The tail was significantly longer than the body, averaging 2.5 times the length (Table 4). The tiny transparent pill-like nuclei appeared within the tail, mostly in the tail’s central axis and spreading sparsely from the anterior to the posterior of the tail. These nuclei were easily noticeable in the stained specimens. The translucent, transversely striated, dilated tegument enveloped the anterior tail portion with an average of 1/3 the tail length. The dorso-ventral finfold was like a thin translucent membrane that materializes bilaterally along the tail edges. The finfold location slightly overlapped on the posterior end of the anterior tail portion’s tegument until it slightly passes the tail posterior tip.

**Table 4:** Comparison of measured metrical data (in μm) of *Opisthorchis viverrini* s.l. cercariae from different snail host taxa in different habitats at different areas between the present study and previous studies.

Source	Present study	Boonmekam et al. [7]	Ngern-klun et al. [36]	Scholz et al. [47]	Wykoff et al. [63]
Study area	BMR	Cambodia	NTH	Lao PDR	NETH
Habitat type	CNS	NST-STWB	Rice field	Rice field	Unspecified
Source of cercariae	*B. s. siamensis*	NST-BS	*B. funiculata*	BSG	BSG
Examination method	Shedding	NST-MOC	Shedding	NST-MOC	Shedding
Type of specimen	Fixed	Fixed	N/A	Fixed	Fixed
Fixation method	10% NBF	N/A	N/A	4% GAiCB	Hot formalin
Dyeing method	Unstained	Unstained + NR	N/A	Gold coating	N/A
Type of microscope	CLM	CLM	CLM	SEM	N/A
No. of specimens	20	10	N/A	N/A	N/A
Body width (BW)	87 ± 8	–	58 ± 12	–	75 ± N/A
	(72–96)	(31–42)	–	(33–68)	(61–96)
Body length (BL)	183 ± 10	–	184 ± 24	–	154 ± N/A
	(167–195)	(50–62)	–	(100–250)	(140–183)
Oral sucker width (OSW)	34 ± 2	–	–	–	36 ± N/A
	(30–38)	(8–13)	–	–	(36–37)
Oral sucker length (OSL)	38 ± 3	–	–	–	43 ± N/A
	(34–41)	(9–14)	–	–	(34–51)
Ventral sucker width (VSW)	22 ± 1	–	–	–	–
	(19–24)	(8–12)	–	–	–
Ventral sucker length (VSL)	24 ± 2	–	–	–	–
	(20–26)	(7–13)	–	–	–
Pharynx width (PW)	18 ± 1	–	–	–	–
	(16–20)	(5–8)	–	–	–
Pharynx length (PL)	19 ± 1	–	–	–	–
	(17–21)	(4–7)	–	–	–
Excretory bladder width (EBW)	41 ± 3	–	–	–	–
	(35–46)	(14–18)	–	–	–
Excretory bladder length (EBL)	61 ± 4	–	–	–	–
	(53–67)	(10–16)	–	–	–
Tail width (TW)	37 ± 3	–	30 ± 5	–	26 ± N/A
	(32–41)	–	–	(16–29)	–
Tail length (TL)	455 ± 10	–	417 ± 38	–	392 ± N/A
	(435–471)	(85–150)	–	(360–475)	(350–437)
Transversely striated tail-tegument width (TSTW)	62 ± 3	–	–	–	–
	(58–65)	–	–	–	–
Transversely striated tail-tegument length (TSTL)	173 ± 10	–	–	–	–
	(154–186)	–	–	–	–
Dorso-ventral finfold width (DFW)	34 ± 2	–	–	–	–
	(31–37)	–	–	–	–
Dorso-ventral finfold length (DFL)	325 ± 13	–	–	–	–
	(310–349)	–	–	–	–
Total length	624 ± 20	–	–	–	532 ± N/A
	(593–651)	–	–	–	(490–565)

Abbreviations: NTH = northern Thailand; NETH = northeastern Thailand; NST-STWB = no specified type of standing-water body (rice field, seasonal lake or pond); NST-BS = no specified taxon of *B. siamensis*; BSG = *B. s. goniomphalos*; NST-MOC = no specified method to obtain the cercariae; 4% GAiCB = 4% glutaraldehyde in cacodylate buffer; CLM = compound light microscope; SEM = scanning electron microscope. The insertion of single hyphenation (–) without numbers on either side and N/A means that data have not been specified.

When comparing the NR color intensity on the alive *O. viverrini* s.l. cercariae structures, most structures within the body were successfully dyed but varied in color intensity, which helped to distinguish these structures, especially the cystogenous glands. Contrarily, the tail and the structures within and on it, particularly the dorso-ventral finfold, were unsuccessfully dyed, except the somewhat successfully dyed tail nuclei. Data from 17 quantitative morphological characteristics of the *O. viverrini* s.l. cercariae were enumerated and compared to previous studies in Table 4.

### Molecular analyses

**Figure 5:**
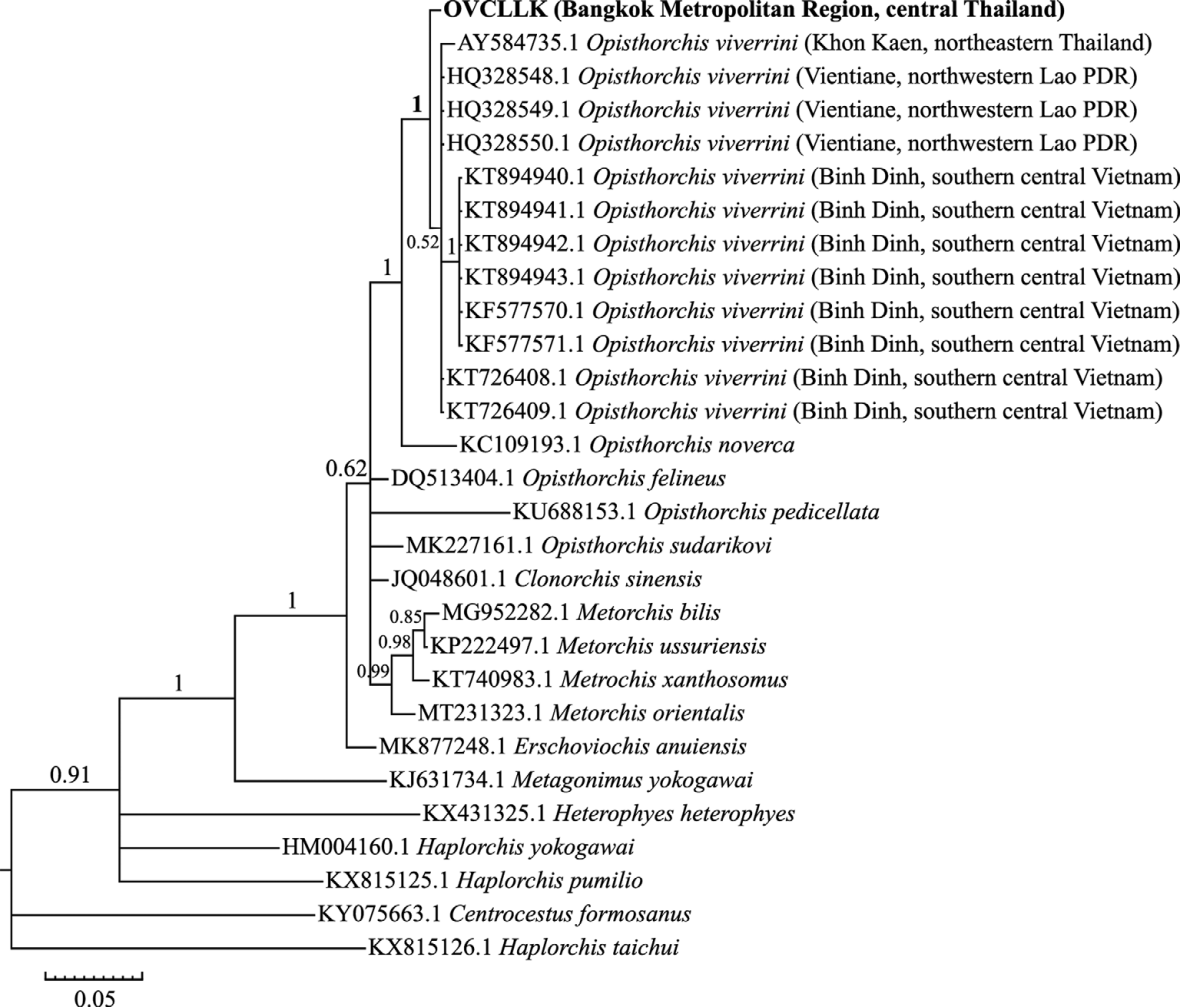
Phylogenetic tree based on the Bayesian analysis of ITS2 sequences. The bold taxon (OVCLLK18) represents the *Opisthorchis viverrini* s.l. cercariae in this study. Numbers at each node display the Bayesian posterior probability value. The branch length is indicated by a scale bar measured in the number of nucleotide substitutions per site.

The morphologically verified *O. viverrini* s.l. cercariae from all four infected snails were *O. viverrini* s.l. species based on the presence of a DNA band from PCR products of all four cercarial sources in a position parallel to the 330 bp specific pOV-A6 DNA band from the PCR products of *O. viverrini* adults (Fig. 4, which displays the example of the one-time gel electrophoresis result from a total of four detection tests). Additionally, phylogenetic analysis of the digeneans according to 394 bp (after the multiple alignment and improvement-by-trimming steps) of the ITS2 region of a representative sample from a total of four samples, was conducted with the BI method for species corroboration among the current and contemporaneous *O. viverrini* s.l. and other *Opisthorchis* species. The other opisthorchiid and heterophyid taxa were considered as the outgroup, along with an inspection of the relationships thereof. The Bayesian phylogram (Fig. 5) established a monophyletic group of the current *O. viverrini* (OVCLLK18) and the contemporaneous *O. viverrini* s.l. (see Table 1 and Fig. 5) with the maximum support value (BPP = 1), which phylogenetically substantiated that OVCLLK18 was of the *O. viverrini* s.l. species.

## Discussion

*Bithynia s. siamensis* snails were not found in five BMR localities, although the BMR CNS provides plenty of habitats and spreading routes for aquatic animals. The urban environment of southern and central Bangkok might not suit the lifestyle of *B. s. siamensis* [56]. Based on our observations, the strong waves created by frequent high-speed motorboats in Pak Kret may significantly disrupt the growth, reproduction, and lifecycle of *B. s. siamensis* [19]. In Phra Samut Chedi and Mueang Samut Prakan, *B. s. siamensis* might be unable to endure the high-salinity water caused by seawater intrusions from the Gulf of Thailand [51]. These reveal a considerably low risk of *O. viverrini* s.l. infections to fish hosts and, subsequently, humans in these four localities. Interestingly, the four *O. viverrini* s.l.-detected localities had a high frequency of snail encounters, which might indicate an encountering risk between snail host and *O. viverrini* s.l. eggs [48].

Interestingly, the prevalences of *O. viverrini* s.l. infections in *Bithynia* snails from the CNS in BMR of this study were lower than in previous studies. The 1.6% *O. viverrini* s.l. prevalence in *B. s. siamensis* from standing-water bodies in Bangna, Bangkok was reported four decades ago [59]. However, most Bangna areas have changed from agricultural land to urbanized areas [10] that may not suit *B. s. siamensis*. Ranges of the mean, total, and overall prevalences of *O. viverrini* s.l. infections in *Bithynia* spp. from various standing-water habitats in northeastern Thailand, Cambodia, Lao PDR, and southern Vietnam were 1.39–3.04%, 0.0–6.8%, and 0.11–1.73%, respectively [7, 9, 17, 27, 28, 30, 32, 35, 59]. With respect to the populations living in these opisthorchiasis endemic areas, their lifestyles may be associated with higher-prevalence causes than in the present study. Unhygienic defecation and consumption of undercooked fishes have been reported in some populations, along with high *O. viverrini* s.l. prevalences in these previously studied areas [16, 32, 40, 60]. Interestingly, there seem to be consistencies among the relatively high *O. viverrini* s.l. infections in *Bithynia* snails, cyprinoid fishes, and humans in northern and northeastern Thailand [27, 36, 41, 55], Cambodia [7, 12, 32], Lao PDR [11, 27, 60], and Vietnam [16, 17]. Moreover, high to very high *O. viverrini* s.l. prevalences (30.92–53.1%) were reported in cats in these areas [2, 60]. These factors possibly lead to continuous transmissions and accumulations of *O. viverrini* s.l. in ecosystems, which might be reflected by the high infection in *Bithynia* snails. Similarly, in the BMR, unsanitary excretion by cats and dogs was scarcely reported [21, 52], along with lower infection rates (1.6%) [21] and very low prevalence in humans that is steadily decreasing [55].

Another cause of very low *O. viverrini* s.l. infection in this study might relate to the unique characteristic of inland aquatic ecosystems possessing the CNS in BMR. The highest *O. viverrini* s.l. prevalences in *B. s. goniomphalos* collected from standing-water habitats have been reported in the cool–dry season in northeastern Thailand [9, 28, 35] that may be due to the decreased water content, leading to high densities of snails [54] and *O. viverrini* s.l. eggs concurrently [35]. In contrast, a similar situation might hardly occur in the CNS in BMR since it is supported continuously by enormous water masses from the upper and middle Chao Phraya River systems [33, 50]. Although the highest prevalence in snails was reported in the wet season in northeastern Thailand [13], the greater amount of water entering the CNS [33, 50] from extreme rainfalls in BMR [31] may lead to dilutions of *O. viverrini* s.l. eggs and cercariae, resulting in a reduced chance of *O. viverrini* s.l. eggs being ingested, and cercariae fish-finding success. Furthermore, water currents in the CNS might push the *O. viverrini* s.l. eggs and cercariae away from *B. s. siamensis* [37, 58] and fish hosts [6, 64], especially in the wet season when water flow velocity increases [22, 43]. Moreover, the spontaneous water currents might complicate host finding [20] and reduce the life expectancy of *O. viverrini* s.l. cercariae [6], and might kill them at high velocity with high shear turbulences [64]. These factors may disrupt lifecycles and accumulations of *O. viverrini* s.l. in ecosystems that might eventually result in decreases of encountering-frequency and infection of *O. viverrini* s.l. in *B. s. siamensis* in the BMR CNS [6, 22, 31, 33, 43, 58, 64].

In Thailand, BMR has the highest proportion (35.79%) of migrant workers [5]. High *O. viverrini* s.l. prevalences have been reported in workers who migrated from northeastern Thailand [53], Lao PDR, and Cambodia [25]. With respect to migrant workers, there might possibly be migrants infecting *O. viverrini* s.l. in BMR. However, the *O. viverrini* s.l. infection rate in *B. s. siamensis* in the CNS in BMR may remain very low due to dilution [37, 58] and withdrawal effects [22, 43] from canal waters on *O. viverrini* s.l. eggs. Similarly, the nature of works in the major economic sectors, like the construction sector [5] that frequently relocate, might cause discontinuous transmission of *O. viverrini* s.l., leading to very low frequency infections in *B. s. siamensis* in the BMR.

The prevalence of *O. viverrini* s.l. cercariae is relatively lower than Xiphidiocercariae and Cercariaeum cercariae, possibly as these two have the intermediate and definitive hosts live in the same ecosystem [39, 45, 49]. In contrast, based on our observation, the tiny numbers of emerged Cystophorous cercariae from the snail (probably due to the parasite’s overlong size: ~2500 μm) may reduce infection to predatory hosts, unlike *O. viverrini* s.l., parapleurolophocercous, furcocercous, and monostome cercarial types whose intermediate and definitive hosts exist in different ecosystems [23, 39, 45].

The identification of samples in this study based on morphological descriptions [23, 63] and the detection using species-specific primers [62], which were utilized in previous studies [7, 13, 28–30, 35, 42, 57], and the phylogenetic analysis using the available DNA sequence (i.e., ITS2 region) [7, 30, 57] provided consistent species. Interestingly, within the *O. viverrini* s.l. clade, the phylogenetic relationship of the *O. viverrini* s.l. in Khon Kaen in northeastern Thailand is closer to the *O. viverrini* s.l. in Lao PDR and Vietnam than the *O. viverrini* s.l. in BMR, despite BMR and Khon Kaen being in the same country.

The *O. viverrini* s.l. cercariae from this study appeared indistinguishable based on qualitative morphological characteristics of *O. viverrini* s.l. cercariae from *B. s. goniomphalos* [63]. However, the quantitative morphological characteristics of *O. viverrini* s.l. cercariae from this study have most structures that were slightly longer and broader than in most previous studies [36, 47, 63], and more than those described by Boonmekam et al. [7] approximately 2–4 times. The latter difference might be because *O. viverrini* s.l. cercariae are an immature stage [14]. The former variations possibly reflect parasite adaptations in different habitat types and relationships to genetic variation [4, 46]. Data on the additionally measured quantitative morphological characteristics and the qualitative morphological characteristics in this study may facilitate species identification of *O. viverrini* s.l. cercariae.

In conclusion, this study is the first to report on *O. viverrini* s.l. infections in any snail host in an uninvestigated habitat for any digenean infections in Thailand, like the network system of flowing-water bodies (i.e., CNS). It is also the first to provide a comprehensive survey in BMR, and revealed very low *O. viverrini* s.l. prevalence. The study also provides molecularly passed-verified, qualitative and quantitative morphological descriptions and metric measurement guidelines to identify the *O. viverrini* s.l. cercariae. Environmental data, which might correlate with *O. viverrini* s.l. infections, should additionally be assessed comprehensively to apply in developing opisthorchiasis-preventive applications in the future.

## Conflict of interest

The authors declare that they have no conflicts of interest related to this article.

## References

[1] Anucherngchai S, Tejangkura T, Chontananarth T. 2016 Epidemiological situation and molecular identification of cercarial stage in freshwater snails in Chao-Phraya Basin, Central Thailand. Asian Pacific Journal of Tropical Biomedicine, 6(6), 539–545.

[2] Aunpromma S, Kanjampa P, Papirom P, Tangkawattana S, Tangkawattana P, Tesana S, Boonmars T, Suwannatrai A, Uopsai S, Sukon P, Sripa B. 2016 Prevlence and risk factors for *Opisthorchis viverrini* infection among cats and dogs in six districts surrounding the Ubolratana Dam, an endemic area for human opisthorchiasis in Northeastern Thailand. Southeast Asian Journal of Tropical Medicine and Public Health, 47, 1153–1159. 29634175

[3] Barber KE, Mkoji GM, Loker ES. 2000 PCR-RFLP analysis of the ITS2 region to identify *Schistosoma haematobium* and *S. bovis* from Kenya. American Journal of Tropical Medicine and Hygiene, 62(4), 434–440. 10.4269/ajtmh.2000.62.434 11220757

[4] Barnett LJ, Miller TL. 2018 Phenotypic plasticity of six unusual cercariae in nassariid gastropods and their relationships to the Acanthocolpidae and Brachycladiidae (Digenea). Parasitology International, 67(2), 225–232. 29288138 10.1016/j.parint.2017.12.004

[5] Benjamin H. 2019 Thailand Migration Report 2019, Benjamin H, Editor. United Nations Thematic Working Group on Migration in Thailand: Bangkok, Thailand.

[6] Bodensteiner LR, Sheehan RJ, Wills PS, Brandenburg AM, Lewis WM. 2000 Flowing water: an effective treatment for ichthyophthiriasis. Journal of Aquatic Animal Health, 12(3), 209–219.

[7] Boonmekam D, Namchote S, Matsuda H, Kirinoki M, Miyamoto K, Sinuon M, Krailas D. 2017 Morphological and molecular identification of the liver fluke *Opisthorchis viverrini* in the first intermediate host *Bithynia* snails and its prevalence in Kampong Cham Province, Cambodia. Parasitology International, 66(3), 319–323. 28189767 10.1016/j.parint.2017.01.016

[8] Brandt RAM. 1974 The non-marine aquatic mollusca of Thailand. Archiv für Molluskenkunde, 105, 1–423.

[9] Brockelman WY, Upatham ES, Viyanant V, Ardsungnoen S, Chantanawat R. 1986 Field studies on the transmission of the human liver fluke, *Opisthorchis viverrini*, in northeast Thailand: population changes of the snail intermediate host. International Journal for Parasitology, 16(5), 545–552. 3781736 10.1016/0020-7519(86)90091-3

[10] Carnegie Mellon University CREATE lab. 2019 Google Earth Engine – Google Earth Timelapse. [cited; Available from: https://earthengine.google.com/timelapse/.

[11] Chai J-Y, Lee S-H, Rim H-J, Sohn W-M, Phommasack B. 2019 Infection status with zoonotic trematode metacercariae in fish from Lao PDR. Acta Tropica, 199, 105100. 31404522 10.1016/j.actatropica.2019.105100

[12] Chai J-Y, Sohn W-M, Na B-K, Yong T-S, Eom KS, Yoon C-H, Hoang E-H, Jeoung H-G, Socheat D. 2014 Zoonotic trematode metacercariae in fish from Phnom Penh and Pursat, Cambodia. Korean Journal of Parasitology, 52(1), 35–40. 10.3347/kjp.2014.52.1.35 PMC3948991 24623879

[13] Chaiyasaeng M, Pechdee P, Sereewong C, Suwannatrai A, Laha T, Tesana S. 2019 Effects of aestivation on survival of *Bithynia siamensis goniomphalos* snails and the infection of *Opisthorchis viverrini* in the irrigation area of wet- and dry-season rice paddy. Acta Tropica, 192, 55–60. 30659807 10.1016/j.actatropica.2019.01.014

[14] Coelho LHL, Guimarães MP, Lima WS. 2008 Influence of shell size of *Lymnaea columella* on infectivity and development of *Fasciola hepatica*. Journal of Helminthology, 82(1), 77–80. 18275634 10.1017/S0022149X08873579

[15] Daniels RA. 2001 Untested assumptions: the role of canals in the dispersal of Sea Lamprey, Alewife, and other fishes in the Eastern United States. Environmental Biology of Fishes, 60(4), 309–329.

[16] Dao TTH, Bui TV, Abatih EN, Gabriël S, Nguyen TTG, Huynh QH, Nguyen CV, Dorny P. 2016 *Opisthorchis viverrini* infections and associated risk factors in a lowland area of Binh Dinh Province, Central Vietnam. Acta Tropica, 157, 151–157. 26872984 10.1016/j.actatropica.2016.01.029

[17] Dao HTT, Dermauw V, Gabriël S, Suwannatrai A, Tesana S, Nguyen GTT, Dorny P. 2017 *Opisthorchis viverrini* infection in the snail and fish intermediate hosts in Central Vietnam. Acta Tropica, 170, 120–125. 28242064 10.1016/j.actatropica.2017.02.028

[18] Esch GW, Barger MA, Fellis KJ. 2002 The transmission of digenetic trematodes: style, elegance, complexity. Integrative and Comparative Biology, 42(2), 304–312. 21708722 10.1093/icb/42.2.304

[19] Gabel F, Lorenz S, Stoll S. 2017 Effects of ship-induced waves on aquatic ecosystems. Science of the Total Environment, 601–602, 926–939. 10.1016/j.scitotenv.2017.05.206 28582738

[20] Haas W, Granzer M, Brockelman CR. 1990 *Opisthorchis viverrini*: finding and recognition of the fish host by the cercariae. Experimental Parasitology, 71(4), 422–431. 2226703 10.1016/0014-4894(90)90068-n

[21] Hinz E. 1980 Intestinal helminths in Bangkok stray dogs and their role in public health. Zentralblatt für Bakteriologie, Mikrobiologie und Hygiene. 1. Abt. Originale B, Hygiene, 171, 79–85. 7435002

[22] Jiménez B. 2007 Helminth ova removal from wastewater for agriculture and aquaculture reuse. Water Science and Technology, 55, 485–493. 17305174 10.2166/wst.2007.046

[23] Kaewkes S. 2003 Taxonomy and biology of liver flukes. Acta Tropica, 88(3), 177–186. 14611872 10.1016/j.actatropica.2003.05.001

[24] Kaewpitoon N, Kaewpitoon S-J, Pengsaa P, Sripa B. 2008 *Opisthorchis viverrini*: the carcinogenic human liver fluke. World Journal of Gastroenterology, 14(5), 666–674. 18205254 10.3748/wjg.14.666 PMC2683991

[25] Kaewpitoon SJ, Sangwalee W, Kujapun J, Norkaew J, Wakkhuwatapong P, Chuatanam J, Loyd RA, Pontip K, Ponphimai S, Chavengkun W, Padchasuwan N, Meererksom T, Tongtawee T, Matrakool L, Panpimanmas S, Kaewpitoon N. 2018 *Opisthorchis viverrini* infection among migrant workers in Nakhon Ratchasima province, Thailand, indicates continued need for active surveillance. Tropical Biomedicine, 35(2), 453–463. 33601819

[26] Khuntikeo N, Titapun A, Loilome W, Yongvanit P, Thinkhamrop B, Chamadol N, Boonmars T, Nethanomsak T, Andrews RH, Petney TN, Sithithaworn P. 2018 Current perspectives on opisthorchiasis control and cholangiocarcinoma detection in Southeast Asia. Frontiers in Medicine, 5, 117. 29765958 10.3389/fmed.2018.00117 PMC5938629

[27] Kiatsopit N, Sithithaworn P, Saijuntha W, Boonmars T, Tesana S, Sithithaworn J, Petney TN, Andrews RH. 2012 Exceptionally high prevalence of infection of *Bithynia siamensis goniomphalos* with *Opisthorchis viverrini* cercariae in different wetlands in Thailand and Lao PDR. American Journal of Tropical Medicine and Hygiene, 86(3), 464–469. 10.4269/ajtmh.2012.11-0217 PMC3284363 22403318

[28] Kiatsopit N, Sithithaworn P, Kopolrat K, Andrews RH, Petney TN. 2014 Seasonal cercarial emergence patterns of *Opisthorchis viverrini* infecting *Bithynia siamensis goniomphalos* from Vientiane Province, Lao PDR. Parasites & Vectors, 7(1), 551. 25442515 10.1186/s13071-014-0551-1 PMC4258299

[29] Kim CS, Echaubard P, Suwannatrai A, Kaewkes S, Wilcox BA, Sripa B. 2016 Seasonal and spatial environmental influence on *Opisthorchis viverrini* intermediate hosts, abundance, and distribution: insights on transmission dynamics and sustainable control. PLoS Neglected Tropical Diseases, 10(11), e0005121. 27880787 10.1371/journal.pntd.0005121 PMC5120785

[30] Laoprom N, Kiatsopit N, Sithithaworn P, Kopolrat K, Namsanor J, Andrews RH, Petney TN. 2016 Cercarial emergence patterns for *Opisthorchis viverrini* sensu lato infecting *Bithynia siamensis goniomphalos* from Sakon Nakhon Province, Thailand. Parasitology Research, 115(9), 3313–3321. 27154765 10.1007/s00436-016-5089-z

[31] Limjirakan S, Limsakul A, Sriburi T. 2009 Trends in temperature and rainfall extremes in Bangkok Metropolitan area, in 21st Conference on Climate Variability and Change. American Meteorological Society: Phoenix, Arizona, USA p. 1–8.

[32] Miyamoto K, Kirinoki M, Matsuda H, Hayashi N, Chigusa Y, Sinuon M, Chuor CM, Kitikoon V. 2014 Field survey focused on *Opisthorchis viverrini* infection in five provinces of Cambodia. Parasitology International, 63(2), 366–373. 24342554 10.1016/j.parint.2013.12.003

[33] Molle F. 2002 The closure of the Chao Phraya River Basin in Thailand: its causes, consequences and policy implications, in Asian irrigation in transition–responding to the challenges ahead, 22–23 April 2002 workshop. Asian Institute of Technology: Bangkok, Thailand, pp. 1–16.

[34] Myers B. 2018 Drought Impacts to Freshwater Ecosystems in the U.S. Caribbean. [cited 2020 September 23]; Available from: https://www.usgs.gov/land-resources/climate-adaptation-science-centers/drought-impacts-freshwater-ecosystems-us-caribbean.

[35] Namsanor J, Sithithaworn P, Kopolrat K, Kiatsopit N, Pitaksakulrat O, Tesana S, Andrews RH, Petney TN. 2015 Seasonal transmission of *Opisthorchis viverrini* sensu lato and a lecithodendriid trematode species in *Bithynia siamensis goniomphalos* snails in northeast Thailand. American Journal of Tropical Medicine and Hygiene, 93(1), 87–93. 10.4269/ajtmh.14-0639 PMC4497911 25918210

[36] Ngern-klun R, Sukontason KL, Tesana S, Sripakdee D, Irvine KN, Sukontason K. 2006 Field investigation of *Bithynia funiculata*, intermediate host of *Opisthorchis viverrini* in northern Thailand. Southeast Asian Journal of Tropical Medicine and Public Health, 37(4), 662–672. 17121291

[37] Ngoen-klan R, Piangjai S, Somwang P, Moophayak K, Sukontason K, Sukontason KL, Sampson M, Irvine K. 2010 Emerging helminths infection in snails and cyprinoid fish in sewage treatment wetlands waters in Cambodia. Asian Journal of Water, Environment and Pollution, 7, 13–21.

[38] Olivier L, Schneiderman M. 1956 A method for estimating the density of aquatic snail populations. Experimental Parasitology, 5(2), 109–117. 13317935 10.1016/0014-4894(56)90008-x

[39] Olsen OW. 1974 Animal parasites: their life cycles and ecology. New York, United States: Dover Publications Inc 564 p.

[40] Pengput A, Schwartz DG. 2020 Risk factors for *Opisthorchis viverrini* infection: a systematic review. Journal of Infection and Public Health, 13(9), 1265–1273. 32564936 10.1016/j.jiph.2020.05.028

[41] Pinlaor S, Onsurathum S, Boonmars T, Pinlaor P, Hongsrichan N, Chaidee A, Haonon O, Limviroj W, Tesana S, Kaewkes S, Sithithaworn P. 2013 Distribution and abundance of *Opisthorchis viverrini* metacercariae in cyprinid fish in Northeastern Thailand. Korean Journal of Parasitology, 51(6), 703–710. 10.3347/kjp.2013.51.6.703 PMC3916461 24516277

[42] Prasopdee S, Kulsantiwong J, Piratae S, Khampoosa P, Thammasiri C, Suwannatrai A, Laha T, Grams R, Loukas A, Tesana S. 2015 Temperature dependence of *Opisthorchis viverrini* infection in first intermediate host snail, *Bithynia siamensis goniomphalos*. Acta Tropica, 141, 112–117. 24161535 10.1016/j.actatropica.2013.10.011

[43] Reinoso R, Torres LA, Bécares E. 2008 Efficiency of natural systems for removal of bacteria and pathogenic parasites from wastewater. Science of the Total Environment, 395(2–3), 80–86. 10.1016/j.scitotenv.2008.02.039 18374393

[44] Sacha S, Suwit T, Ladawan K, Surapol P. 2001 Thailand’s water vision: a case study. [cited 2020 September 23]; Available from: http://www.fao.org/3/AB776E/ab776e04.htm.

[45] Schell SC. 1970 How to know the trematodes. Dubuque, Iowa: Wm. C. Brown Company 354 p.

[46] Schneider RF, Meyer A. 2017 How plasticity, genetic assimilation and cryptic genetic variation may contribute to adaptive radiations. Molecular Ecology, 26(1), 330–350. 27747962 10.1111/mec.13880

[47] Scholz T, Ditrich O, Giboda M. 1992 Study on the surface morphology of the developmental stages of the liver fluke, *Opisthorchis viverrini* (Trematoda: Opisthorchiidae). Annales de Parasitologie Humaine et Comparée, 67, 82–90. 1290379 10.1051/parasite/199267382

[48] Schulze TL, Jordan RA, Schulze CJ, Mixson T, Papero M. 2005 Relative encounter frequencies and prevalence of selected *Borrelia*, *Ehrlichia*, and *Anaplasma* Infections in *Amblyomma americanum* and *Ixodes scapularis* (Acari: Ixodidae) ticks from Central New Jersey. Journal of Medical Entomology, 42(3), 450–456. 15962799 10.1093/jmedent/42.3.450

[49] Shimazu T. 2016 Digeneans parasitic in freshwater fishes (Osteichthyes) of Japan VI. Lissorchiidae. Bulletin of the National Museum of Nature and Science. Series A, Zoology, 42(1), 1–22.

[50] Souris M. 2018 Thailand rivers and streams. Marseille, French: Institute of Research for Development (IRD).

[51] Stoecker F, Babel MS, Gupta AD, Rivas AA, Evers M, Kazama F, Nakamura T. 2013 Hydrogeochemical and isotopic characterization of groundwater salinization in the Bangkok aquifer system, Thailand. Environmental Earth Sciences, 68(3), 749–763.

[52] Sukthana Y, Kaewkungwal J, Jantanavivat C, Lekkla A, Chiabchalard R, Aumarm W. 2003 *Toxoplasma gondii* antibody in Thai cats and their owners. Southeast Asian Journal of Tropical Medicine and Public Health, 34(4), 733–738. 15115080

[53] Suwannahitatorn P, Klomjit S, Naaglor T, Taamasri P, Rangsin R, Leelayoova S, Mungthin M. 2013 A follow-up study of *Opisthorchis viverrini* infection after the implementation of control program in a rural community, central Thailand. Parasite Vectors, 6, 188. 10.1186/1756-3305-6-188 PMC3689608 23786863

[54] Suwannatrai A, Suwannatrai K, Haruay S, Piratae S, Thammasiri C, Khampoosa P, Kulsantiwong J, Prasopdee S, Tarbsripair P, Suwanwerakamtorn R, Sukchan S, Boonmars T, Malone JB, Kearney MT, Tesana S. 2011 Effect of soil surface salt on the density and distribution of the snail *Bithynia siamensis goniomphalos* in northeast Thailand. Geospatial Health, 5(2), 183–190. 21590668 10.4081/gh.2011.170

[55] Suwannatrai A, Saichua P, Haswell M. 2018 Chapter two – epidemiology of *Opisthorchis viverrini* infection. Advances in Parasitology, 101, 41–67. 29907255 10.1016/bs.apar.2018.05.002

[56] Tchakonte S, Ajeagah GA, Diomande D, Camara AI, Ngassam P. 2014 Diversity, dynamic and ecology of freshwater snails related to environmental factors in urban and suburban streams in Douala-Cameroon (Central Africa). Aquatic Ecology, 48(4), 379–395.

[57] Thaenkham U, Blair D, Nawa Y, Waikagul J. 2012 Families Opisthorchiidae and Heterophyidae: Are they distinct? Parasitology International, 61(1), 90–93. 21740979 10.1016/j.parint.2011.06.004

[58] Tiev V, Mongtoeun Y, Saneth V, Kim I, Thammarat K. 2010 Efficiency of Phnom Penh’s natural wetlands in treating wastewater discharges. Asian Journal of Water, Environment and Pollution, 7(3), 39–48.

[59] Upatham ES, Sukhapanth N. 1980 Field studies on the bionomics of *Bithynia siamensis siamensis* and the transmission of *Opisthorchis viverrini* in Bangna, Bangkok, Thailand. Southeast Asian Journal of Tropical Medicine and Public Health, 11(3), 355–358. 7444576

[60] Vonghachack Y, Odermatt P, Taisayyavong K, Phounsavath S, Akkhavong K, Sayasone S. 2017 Transmission of *Opisthorchis viverrini*, *Schistosoma mekongi* and soil-transmitted helminthes on the Mekong Islands, Southern Lao PDR. Infectious Diseases of Poverty, 6(1), 131. 28866984 10.1186/s40249-017-0343-x PMC5582398

[61] Westcot DW. 1997 Quality control of wastewater for irrigated crop production. Chapter 2 – Health risks associated with wastewater use 1997. [Available from: http://www.fao.org/3/w5367e/w5367e04.htm]. Accessed date: September 23, 2020.

[62] Wongratanacheewin S, Pumidonming W, Sermswan RW, Maleewong W. 2001 Development of a PCR-based method for the detection of *Opisthorchis viverrini* in experimentally infected hamsters. Parasitology, 122(Pt 2), 175–180. 11272648 10.1017/s0031182001007235

[63] Wykoff DE, Harinasuta C, Juttijudata P, Winn MM. 1965 *Opisthorchis viverrini* in Thailand – the life cycle and comparison with *O. felineus*. Journal of Parasitology, 51, 207–214. 14275209

[64] Xue Z, Gebremichael M, Ahmad R, Weldu ML, Bagtzoglou AC. 2011 Impact of temperature and precipitation on propagation of intestinal schistosomiasis in an irrigated region in Ethiopia: suitability of satellite datasets. Tropical Medicine & International Health, 16(9), 1104–1111. 21767333 10.1111/j.1365-3156.2011.02820.x

